# Implementation and Optimization of a Dual-confocal Autofocusing System

**DOI:** 10.3390/s20123479

**Published:** 2020-06-19

**Authors:** Chia-Ming Jan, Chien-Sheng Liu, Jyun-Yi Yang

**Affiliations:** 1 Metal Industries Research and Development Centre, Kaohsiung City 81160, Taiwan; cmjan@mail.mirdc.org.tw; 2 Department of Mechanical Engineering, National Cheng Kung University, Tainan City 70101, Taiwan; 3 Department of Mechanical Engineering, National Chung Cheng University, Chiayi County 62102, Taiwan; bonbonjn@hotmail.com

**Keywords:** autofocusing, autofocusing system, dual-confocal, confocal, optical optimization

## Abstract

This paper describes the implementation and optimization of a dual-confocal autofocusing system that can easily describe a real-time position by measuring the response signal (i.e., intensity) of the front and the rear focal points of the system. This is a new and systematic design strategy that would make it possible to use this system for other applications while retrieving their characteristic curves experimentally; there is even a good chance of this technique becoming the gold standard for optimizing these dual-confocal configurations. We adopt two indexes to predict our system performance and discover that the rear focal position and its physical design are major factors. A laboratory-built prototype was constructed and demonstrated to ensure that its optimization was valid. The experimental results showed that a total optical difference from 150 to 400 mm significantly affected the effective volume of our designed autofocusing system. The results also showed that the sensitivity of the dual-confocal autofocusing system is affected more by the position of the rear focal point than the position of the front focal point. The final optimizing setup indicated that the rear focal length and the front focal length should be set at 200 and 100 mm, respectively. In addition, the characteristic curve between the focus error signal and its position could successfully define the exact position by a polynomial equation of the sixth order, meaning that the system can be straightforwardly applied to an accurate micro-optical auto-focusing system.

## 1. Introduction

Due to its good reliability, high throughput, and relatively low cost, machine vision systems are an attractive solution for the inspection process in automated mass production lines. In practice, such systems always need a highly precise autofocusing capability to obtain sufficiently sharp images of the object of interest [1,2,3,4]. Autofocusing systems have been widely applied in recent decades in a variety of industrial manufacturing and measurement fields, such as in cellphone camera modules, automatically available optical inspection, and dynamic tracking systems [4]. In brief, many autofocusing systems have been developed, and these autofocusing systems can be broadly classified as image-based methods [5,6,7,8,9,10,11,12,13,14,15,16] and optics-based methods [17,18,19,20,21,22,23,24,25,26,27,28,29,30]. Both need to be driven by moving motors to achieve the function of autofocusing, which limits the possibility of their direct implementation for in-line inspection.

In an image-based autofocusing system, the position of the focusing objective lens is determined by capturing real images through a Charge-coupled Device (CCD) or Complementary Metal-Oxide-Semiconductor (CMOS) device with the use of complex image processing. The performance of the image-based autofocusing system depends on the sharpness of the captured images or the image spatial frequency function, which is used to calculate the focus value (FV) in the system. By hill-climbing searching, determining the peak value can help to determine the required focus based on a simple configuration, which is easy to handle and stable but rather time-consuming and has a very limited effective depth of focus. We have found that many research articles [31,32,33,34,35,36,37] have investigated the improvement of the algorithm using software and tried to make the application of the technique to the product line possible.

In the optics-based autofocusing method, the triangle geometric and dual-confocal configurations are widely used in the system. The triangle geometric technologies are well-known optical methods which are applied in many inspection instruments [38,39,40]. Our research group has published many methods to improve the focusing accuracy and response of the optics-based autofocusing systems with triangle geometric technologies [27,28,29,30].

Accordingly, we claim to have developed a novel and easily constructed autofocusing system by adopting a dual-confocal configuration. By utilizing a differential mode, we deal with the spatial optical intensity distribution from the rear and the front focal planes, retrieving two focus error signals. Furthermore, our system can directly retrieve the location of the specific focal plane. Meanwhile, we focus on an implementation in this paper in combination with an optimization method based on the optical autofocusing system. The commercially available software (ZEMAX) was used to verify our experimental setup. The system has the significant benefit of high accuracy, a fast response time, and the retrieval of the exact moving direction while tracking the front and rear focal planes, and there is tremendous potential for applying our system in mini-optomechanics with the required rapid autofocusing technique. Overall, we not only demonstrate the laboratory-built prototype in our study, but also show numerical analyses employed to determine the optimal design parameters of our proposed dual-confocal autofocusing system.

## 2. Methods

The image-based autofocusing system, which captures real images through CCD or CMOS devices with the cooperation of complex image processing, is a classic and well-known technique. Calculating the sharpness of captured images or the FV independently of the image spatial frequency function in the system can indicate the position of the focusing objective lens while in-focus. Chen et al. [7] first discussed an image-based autofocusing system and determined the peak value, which could help to determine focusing based on a simple configuration by hill-climbing searching, which is easy to handle and stable; however, it is rather time-consuming and has a very limited effective depth of focus. They adopted the discrete wavelet transform (DWT) method to perform the sharpness measurement and for further validation.

The fully digital autofocusing (FDAF) method was shown by Jeon et al. [31] to obtain a fast and precise autofocusing module by searching the focusing area automatically in cooperation with the point spread function (PSF). In 2011, Koh et al. claimed a configuration adopting two low-pass filters and double apertures based on the capture of two monochromic images to be able to denoise and distinguish its defocus direction and position through the variance of gradient magnitude (VGM) [32].

In 1993, Yamada et al. [33] developed a patent including a new configuration which was able to resolve the problem of distinguishing the rear focusing beam from the front one under a high-power zoom lens based on the creation of an optical difference by utilizing a switch device and a prism. They only needed to capture these two images to obtain sufficient information, while the exact focal plane was located between the front focusing beam and the rear focusing beam. This represented almost the first concept of the dual-confocal autofocusing system and easily handled the determination of the focusing position and its moving direction.

**Figure 1:**
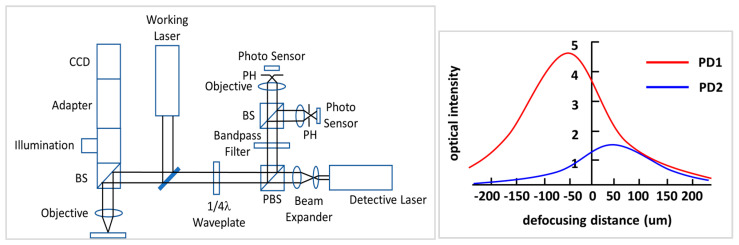
The schematic diagram of the dual-confocal system [41].

The optical-based configuration includes photodetectors (e.g., CCD, CMOS, photomultiplier tube (PMT)) as information detection systems to determine the shape of a laser spot and its intensity to estimate focusing, which is highly precise and quick to perform based on the position error signal (or focus error signal). In 2010, Wang et al. developed a femtosecond laser machining system by adopting the autofocusing module in combination with the dual-confocal system, as shown in Figure 1. The results of their simulations and experiments demonstrated that the values of positioning accuracy and repeatability are both less than ±1.5 μm within the measuring range of ±200 μm based on the respective response intensity of dual near-focusing position [41]. This is also an example of the above-mentioned dual-confocal system configuration being applied for an autofocusing function.

**Figure 2:**
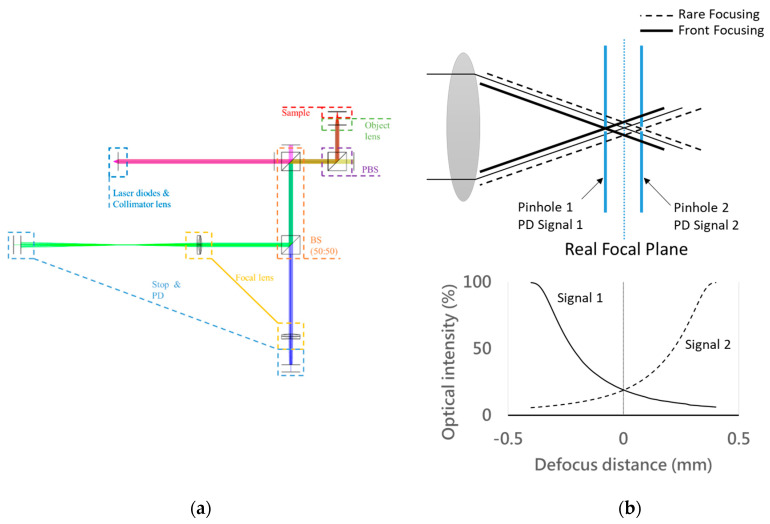
Proposed dual-confocal autofocusing system: (**a**) schematic illustration and (**b**) simulation of the front and the rear focal positions (intensity distribution).

Using the dual-confocal configuration, we propose an optics-based autofocusing system scheme in this paper, as shown in Figure 2a. Meanwhile, the response intensity of the focusing or defocusing position is able to be obtained by two photodetector (PD) signals of the front and rear focal planes, which could be used to calibrate and determine the exact focusing position and its direction. Moreover, we provide a characteristic curve of the focus error signal obtained by the two differential PD signals. Therefore, this optics-based autofocusing system simply utilizes the well-set PDs to retrieve the real-time intensity signal near the focal plane, which can directly indicate the focusing position by simultaneously calculating the merit function of the intensity distribution. Our proposed optics-based autofocusing system has the advantages of being highly precise and having a rapid response time, enabling the module to be easily imported to the production line. Compared with the conventional confocal system, our proposed system can obtain the moving direction information only by retrieving the intensity near the focal plane, i.e., at the exact positions indicated. According to the light intensity distribution of Gaussian focusing, the two parts of the original signal (i.e., intensity) detected from the PDs at the different focal positions will indicate two characteristic curves. The location of the focal plane is indicated by comparing the intersection of the two spatial optical intensity distribution curves with the rear and front defocus distance, respectively, as shown in Figure 2b. Thus, we can obtain the position of the focal plane accompanied with its moving direction, and we only need to calculate the exact optical difference between two light beams for the front focusing and rear focusing. In brief, we propose a novel dual-confocal configuration for an optics-based autofocusing microscope, to be used instead of a conventional confocal system or a centroid knife-edge method. This configuration boasts a simple scheme without redundant moving by trial and error; it has several significant characteristics enabling fast scanning, precise position tracking, and a low cost. Key points related to the above-mentioned methods are shown in Table 1.

## 3. System Implementation

**Figure 3:**
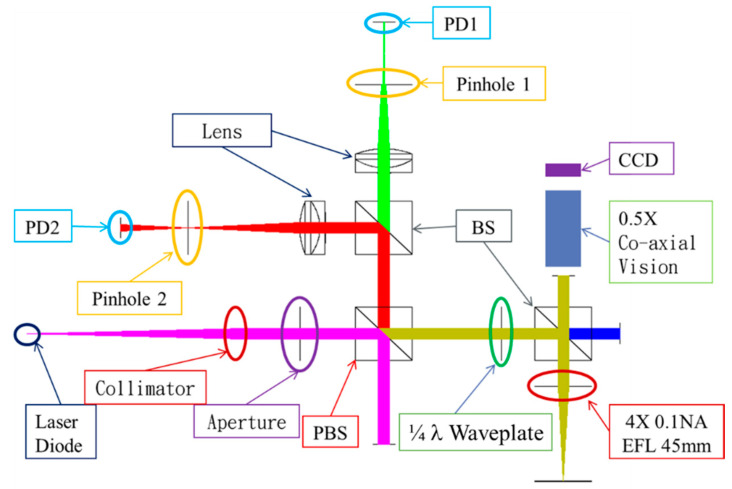
Schematic illustration of our proposed dual-confocal system.

Figure 3 illustrates the brief configuration of our proposed system, including a laser diode, a collimator, the first beam splitter (BS), a microscope module, the second BS, and two pinholes (which set at the front focal plane and at the rear focal plane) that cooperate with the photodetectors (PDs). The light source we adopted was a laser diode (wavelength of 633 nm) made by Thorlabs (HL6501MG). The microscope module we utilized had an objective of 10× (Olympus Co., f = 18 mm) and can cover the scanning area of about 2 mm × 2 mm, which is sufficient an for optics-based autofocusing system with single-point scan application. Table 2 thus shows all key modules adopted in our proposed dual-confocal microscopic system. For comparison with the simulation results (considering the total optical difference from 150 to 400 mm while comparing with the objective of the microscope module), we constructed a prototype of the dual-confocal configuration with a rear focal length of 100 mm and a front focal length of 200 mm. Furthermore, the characteristic curve between focus error signal (FES) and its position can successfully define the exact focusing position by a sixth-order polynomial equation.

## 4. Results

**Figure 4:**
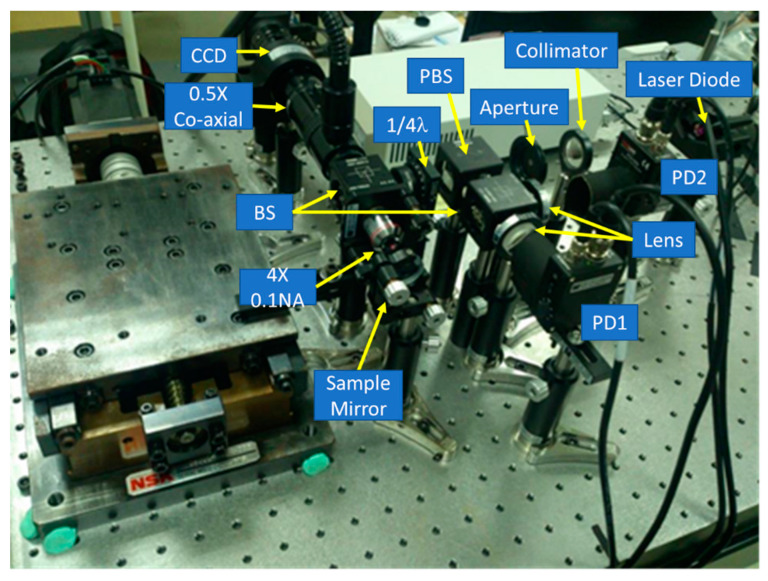
Photograph of the laboratory-built prototype.

**Figure 5:**
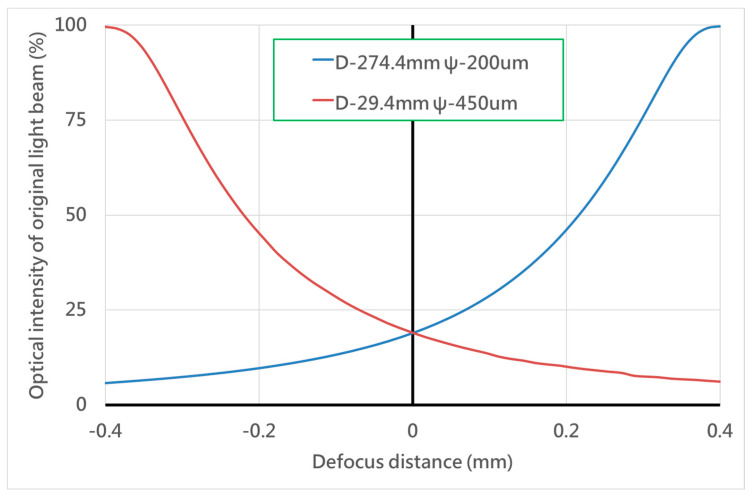
Simulation of the intensity signals from the two photodetectors.

**Figure 6:**
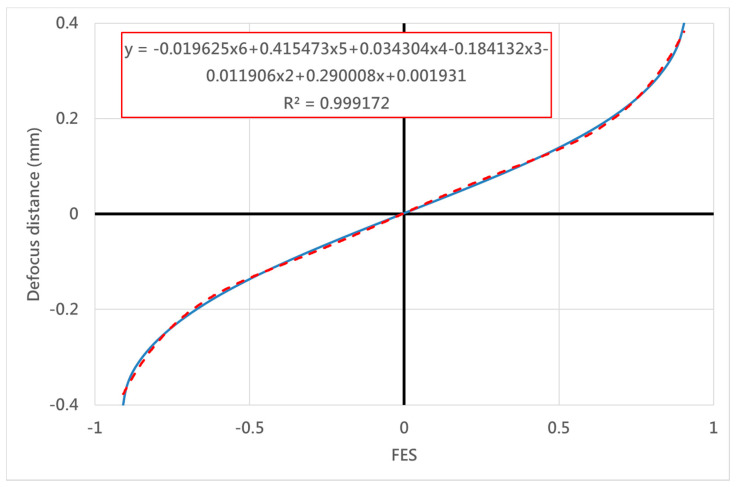
The relationship between the focus error signal (FES) and the defocus distance.

Our proposed configuration was characterized numerically by using the commercially available software (ZEMAX), and then it was verified experimentally using a laboratory-built prototype, as shown in Figure 4. Figure 5 shows the simulation data obtained by the two PDs, which means optical intensity retrieved at the front site and rear site of the focal planes in our proposed system. The both sites setup were chosen on the basis of the similarly symmetrical curves while obtaining single optical intensity distribution versus the defocus distance. The optimal parameters included the four independent parameters, with the primary importance being placed on the rear focal point. The maximum error of our autofocusing system could be evaluated on the basis of several tests (see Table 3). The FES, which can be determined by differential modes of two signals, should be directly be directly dependent on the focusing position, as shown in Figure 6. We constructed an approximation formula refereed to the results obtained from the FES and focusing position. More specifically, the raw data obtained from the PDs can be easily used to calculate and indicate the exact position real-time.

**Figure 7:**
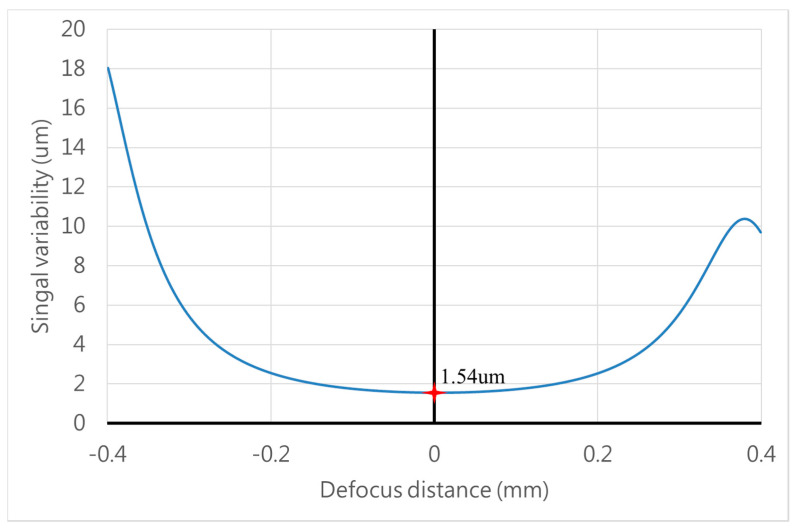
The distribution of signal variability versus defocus distance.

**Figure 8:**
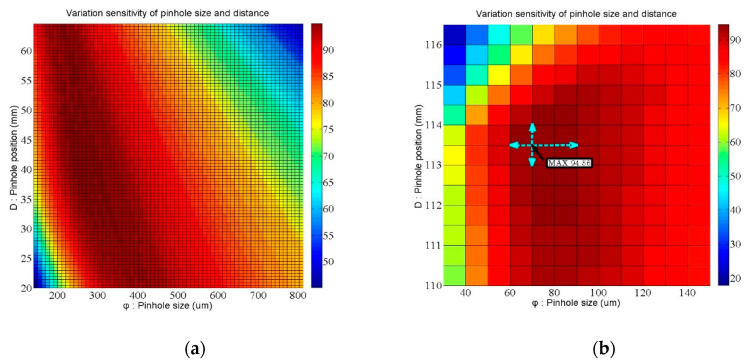
Simulation results of the variation in sensitivity of the autofocusing system depending on (**a**) the position of rear focal point and (**b**) the position of front focal point.

**Figure 9:**
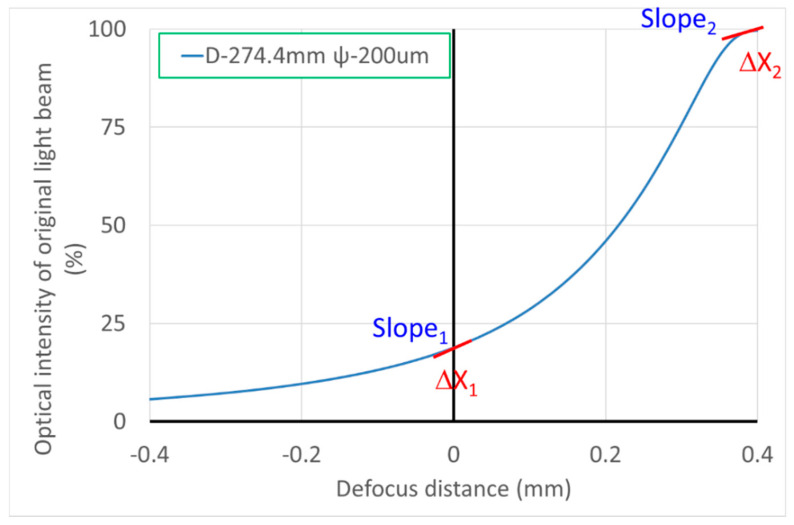
The key index of the characteristic curve in our focusing system.

We demonstrated that the FES calculation can indicate the position through measuring the intensity by two PDs because of the theoretically predictive intensity distribution. Considering the signal variability near the focal position and far away from the focal plane, the minimum FES would be 1.6 μm under the available signal limitation (as shown in Figure 7). Furthermore, the data retrieval performance at the rear focal point is better than at the front one, so we discuss how the relationships between the signal variability (μm) and several parameters (e.g., pinhole position, pinhole size, and effective focal length (EFL)) can be determined by the characteristic curves of the front and rear focal points (as shown in Figure 8). Here, we define the slope and error position as the two set indexes of the characteristic curve, as shown in Figure 9, which represent the signal transfer error and its available linear sensitivity. A low ΔX index when considering the minimum electric signal error is indicative of the potential of our focusing system for highly precise positioning. The slope index values (Slope1 & 2) can be determined from the characteristic curve near the focal plane and away from the focal position, which indicated the linear transformation from the FES calculation well.

**Figure 10:**
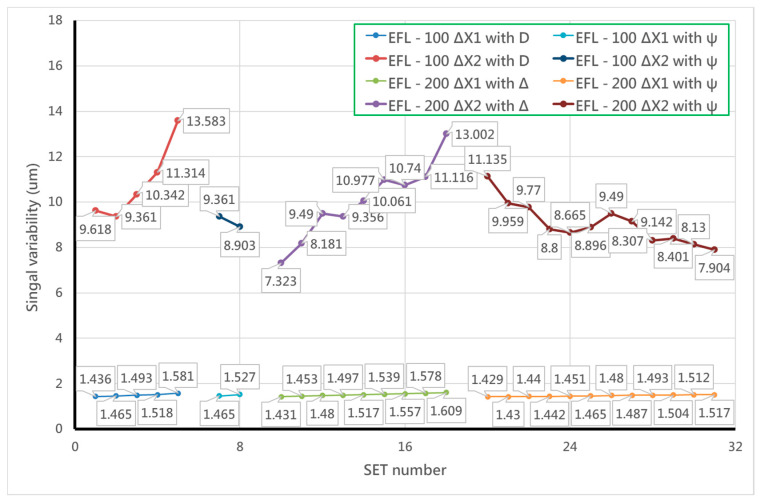
Our simulation of the optimal parameters of the rear focal point.

Table 3 shows our simulation data, including thirty-one sets of the above-mentioned indexes, and the optimal parameters of the rear focal point that we chose in our system. Finally, the optimal pinhole size is 75 μm, the optimal pinhole distance from the objective is 113.5 mm, and the optimal rear focal length is about 100 mm (as shown in Figure 10). Accordingly, the results shown in Figure 10 demonstrate that the measuring sensitivity of the rear focal point is better than that of the front focal point. We determined the optimal setup of the rear focal point first in order to retrieve the ΔX1 and ΔX2 indexes. Furthermore, we also adopted the following optimal parameters for the front focal point: pinhole size of 400 μm, pinhole distance from the objective of 35.6 mm, and rear focal length of about 200 mm. According to Figure 6, a mirror-like curve is constructed with opposite the *x*-axis and the *y*-axis, which is similar to the curve of the rear focal point; this allows the FES to be calculated and its position to be indicated directly.

Our proposed optical configuration was suitably designed and constructed to retrieve an FES from the rear focal plane and another FES from the front focal plane. To indicate the location of the specific focal plane, the relationship between the FES and the defocus distance is adopted and its spatial optical intensity distribution is calculated. In this manuscript, we have proposed and developed a novel and easily constructed autofocusing system.

## 5. Conclusions

This paper successfully describes a new design rule for choosing the optimal system parameters for obtaining the lowest signal variability. For the front and rear focal points, we defined the values of the focal length, pinhole position, and pinhole size in a step-by-step process. Our results also showed how the total optical difference from 150 to 400 mm significantly affected the effective volume of our designed autofocusing system. This finding should be considered carefully when integrating this module into the whole system. We adopted two indexes to predict our system performance and discovered that the rear focal position and its physical design are major factors that directly affect the accuracy of an autofocusing system based on dual-confocal configuration accompanied with sufficient dynamic range.

## Figures and Tables

**Table 1:** Comparison of prior autofocusing system studies.

Development Team	Key Points	Compared with Our Proposed System	Reference
Chen et al.	Image-based Utilizing SOM neural network to calculate the individual FV of the image spatial frequency function Utilizing the DWT method for sharpness measuring	Uses a complex algorithm	[7]
Jeon et al.	Fully digital autofocusing Fast and precise autofocusing Cooperation with point spread function	Has a high-cost image capturing system	[31]
Koh et al.	Adopting two low-pass filters and double apertures Position variance of gradient magnitude (VGM)	Requires a complex algorithm to deal with blurred images	[32]
Yamada et al.	High-power zoom lens Optical difference by utilizing a switch prism	Requires a high-cost and precise positioning for image capturing	[33]
Wang et al.	Response intensity of dual near-focusing position	Without retrieving the information of moving direction near the focal plane	[41]

**Table 2:** Devices used in our proposed system.

Key Parts	Device
Laser Light Source	Laser diode (Thorlabs HL6501MG)
Collimator	Thorlabs LT110P-A (f = 6.24 mm) Thorlabs LT240P-A (f = 8 mm)
Beam Splitter (BS)	Thorlabs CM1-BS013 (50:50)
Polarized Beam Splitter (PBS)	Thorlabs CM1-PBS251
Objective	Olympus Co. (f = 18 mm)
Focusing Lens	Thorlabs AC254-100A Thorlabs AC254-200A
Pinholes	Thorlabs P75S Thorlabs P300S Thorlabs P400S
PD	Thorlabs PDA100A
Co-axial Vision	Navitar 1-6030, 1-60255
Motor	Newport ILS-250HA

**Table 3:** Our simulation of the optimal parameters for the rear focal point.

**SET**	**EFL (mm)**	**Pinhole Position D** **(mm)**	**Pinhole Size ψ (μm)**	**ΔX1 (mm)**	**Slope 1**	**ΔX2 (mm)**	**Slope 2**
1	100	113.5	60	1.436	4.178	9.618	0.624
2			70	1.465	4.095	9.361	0.641
3			75	1.493	4.019	10.342	0.58
4			80	1.518	3.952	11.314	0.53
5			90	1.581	3.795	13.583	0.442
7		113.5	75	1.465	4.095	9.361	0.641
8		114		1.527	3.929	8.903	0.674
**SET**	**EFL (mm)**	**Pinhole Position D** **(mm)**	**Pinhole Size ψ (μm)**	**ΔX1 (mm)**	**Slope 1**	**ΔX2 (mm)**	**Slope 2**
10	200	273.5	140	1.431	4.192	7.323	0.819
11			150	1.453	4.129	8.181	0.733
12			160	1.48	4.055	9.49	0.632
13			170	1.497	4.007	9.356	0.641
14			180	1.517	3.956	10.061	0.596
15			190	1.539	3.9	10.977	0.547
16			200	1.557	3.855	10.74	0.559
17			210	1.578	3.803	11.116	0.54
18			220	1.609	3.728	13.002	0.461
20		270.5	160	1.429	4.197	11.135	0.539
21		271		1.43	4.195	9.959	0.602
22		271.5		1.44	4.168	9.77	0.614
23		272		1.442	4.161	8.8	0.682
24		272.5		1.451	4.134	8.665	0.692
25		273		1.465	4.096	8.896	0.674
26		273.5		1.48	4.055	9.49	0.632
27		274		1.487	4.035	9.142	0.656
28		274.5		1.493	4.019	8.307	0.722
29		275		1.504	3.988	8.401	0.714
30		275.5		1.512	3.969	8.13	0.738
31		276		1.517	3.955	7.904	0.759
